# CRISPR/Cas12a-Assisted Visual Logic-Gate Detection of Pathogenic Microorganisms Based on Water-Soluble DNA-Binding AIEgens

**DOI:** 10.3389/fchem.2021.801972

**Published:** 2022-01-14

**Authors:** Zhe Jiao, Jialing Yang, Xiaojuan Long, Yingfang Lu, Zongning Guo, Yonglin Peng, Xuelin Huang, Yi Yin, Chao Song, Pengfei Zhang

**Affiliations:** ^1^ School of Environment and Civil Engineering, Dongguan University of Technology, Dongguan, China; ^2^ Guangdong Dongguan Ecological and Environmental Monitoring Station, Dongguan, China; ^3^ Huangpu Customs District Technology Center, Dongguan, China; ^4^ Pinete (Zhongshan) Biotechnology Co., Ltd., Zhongshan, China; ^5^ Pearl River Fisheries Research Institute, Chinese Academy of Fishery Sciences, Guangzhou, China,; ^6^ Freshwater Fisheries Research Center, Chinese Academy of Fishery Sciences, Wuxi, China; ^7^ Key Laboratory of Control of Quality and Safety for Aquatic Products, Ministry of Agriculture and Rural Affairs, Beijing, China; ^8^ Guangdong Key Laboratory of Nanomedicine, Shenzhen Engineering Laboratory of Nanomedicine and Nanoformulations, CAS Key Laboratory of Health Informatics, Institute of Biomedicine and Biotechnology, Shenzhen Institute of Advanced Technology, Chinese Academy of Sciences, Shenzhen, China

**Keywords:** CRISPR-Cas12a, pathogenic microorganisms, aggregation-induced emission (AIE), visual detection, logic gate

## Abstract

Here, we developed a rapid, visual and double-checked Logic Gate detection platform for detection of pathogenic microorganisms by aggregation-induced emission luminogens (AIEgens) in combination with Clustered Regularly Interspaced Short Palindromic Repeats (CRISPR)/CRISPR associated (Cas). DNA light-up AIEgens (1,1,2,2-tetrakis[4-(2-bromo-ethoxy) phenyl]ethene, TTAPE) was non-emissive but the emission was turned on in the presence of large amount of DNA produced by recombinase polymerase amplification (RPA). When CRISPR/Cas12a was added, all long-stranded DNA were cut leading to the emission quenched. Thus, a method that can directly observe the emission changes with the naked eye has been successfully constructed. The detection is speedy within only 20 min, and has strong specificity to the target. The result can be judged by Logic Gate. Only when the output signal is (1,0), does it represent the presence of pathogenic microorganisms in the test object. Finally, the method was applied to the detect pathogenic microorganisms in environmental water samples, which proved that this method has high selectivity, specificity and applicability for the detection of pathogenic microorganisms in environmental water samples.

## 1 Introduction

Pathogenic microorganisms can cause human or animal diseases, including viruses, bacteria, fungi, protozoa, worms, etc. They are widely distributed in our environment and cause various human diseases in many cases, which has a serious impact on human health (Feng et al., 2016; Váradi et al., 2017). Among them, *Legionella pneumophila* (*L. pneumophila*) is a typical pathogen which is the pathogen of Legionnaires' disease. It is widely found in freshwater environments such as lakes, streams, air conditioning cooling towers, fountains, and hot spring baths. It can be spread through the air, leading to severe respiratory diseases, with a high case fatality rate (Newton et al., 2010). *Escherichia coli* (*E. coli*) and *Staphylococcus aureus* (*S.aureus*) are the two most common pathogens in the environment (Suaifan et al., 2017). In order to successfully resist infection by these pathogens, timely and accurate detection of multiple pathogenic microorganisms to cut off the source of infection is crucial.

Commonly used methods to detect pathogenic microorganisms include microbial culture (Bouguelia et al., 2013), enzyme linked immunosorbent assay (ELISA) (Nakayama et al., 2010), mass spectrometry (Sauer and Kliem, 2010), polymerase chain reaction (PCR) (Garibyan and Avashia, 2013), flow cytometry (Khan et al., 2010) etc. But these methods have their own limitations, such as time-consuming, cumbersome steps, low sensitivity, long detection time, serious false positives, and large-scale equipment. etc. (Rajapaksha et al., 2018; Zhang et al., 2018) Especially in areas with limited resources, vital development of portable, rapid and costless (Yoo and Lee, 2016). diagnostic methodsis urgently needed.

As a new type of fluorescent material, aggregation-induced emission luminogens (AIEgens) shows weak or negligible emission in dilute solution, but it emits highly in aggregate or solid state (Vendrell et al., 2012; Guan et al., 2017; Chen Y et al., 2018; Liu and Tang, 2019). 1,1,2,2-tetrakis[4-(2-bromo-ethoxy)phenyl]ethene (TTAPE) is a fully water-soluble AIEgens with positive electricity, and can emit a blue fluorescence signal after binding with DNA via electrostatic interaction (Lou et al., 2015; Daher et al., 2016; Zhu et al., 2018). However, If TTAPE combined with recombinase polymerase amplification (RPA) to directly detect, it is easy to cause false positive results. This is because all specificity in the detection process comes from RPA, and the unique primer design of RPA often makes it difficult to avoid the generation of primer dimers, which causes false positive results (Moore and Jaykus, 2017; Lobato and O'Sullivan, 2018; Jiao et al., 2019). Clustered Regularly Interspaced Short Palindromic Repeats (CRISPR)/CRISPR associated (Cas) is a prokaryotic immune system, which is used to resist the invasion of foreign genetic material (Schunder et al., 2013). In recent years, the CRISPR/Cas system has been developed into an efficient gene editing tool that can specifically identify foreign DNA fragments and cut them off (Hille and Charpentier, 2016; Koonin et al., 2017). CRISPR/Cas12a is the second type (V type) CRISPR/Cas, which is guided by a CRISPR RNA (crRNA) that can specifically recognize the DNA sequence and turn on the cleavage activity of Cas12a protein pair (Jinek et al., 2012; Zetsche et al., 2015). Moreover, Cas12a can not only cut the DNA strands bound to itself, but also cut all other DNA that it can find (Chen Y et al., 2018; Li et al., 2018a). In recent years, a series of detection platforms have been developed using the random shearing feature of Cas12a to realize the detection of nucleic acids (Gootenberg et al., 2017; Chen J. S et al., 2018; Li et al., 2018b; Gootenberg et al., 2018). In these reported literature, a fluorescent reporter molecule was required to be prelabled which was costly and complicated. In addition, at least 15 min of reaction time was needed in these process. When the CRISPR/Cas12a technology is combined with TTAPE, the label free interaction of TTAPE with DNA will turn on the fluorescent emission immediately and quenched within 5 min after adding Cas12a, The results accuracy will be improved significantly by dual signal change, which can be analyzed through INHIBIT Gate in Logic Gate. When the output value is (1, 0), pathogenic microorganisms can be detected.

In this study, the TTAPE-CRISPR/Cas12a Logic Gate detection platform was constructed for rapid detection of pathogenic microorganisms. Including *L. pneumophila, E. coli* and *S.aureus*. The advantages of AIE property of TTAPE and the random shearing feature of CRISPR/Cas12a were explored. The platform is also applied to the detection of water samples. It is expected to realize rapid and accurate visual detection of pathogenic microorganisms.

## 2 Results and Discussion

### 2.1 Principles

**FIGURE 1:**
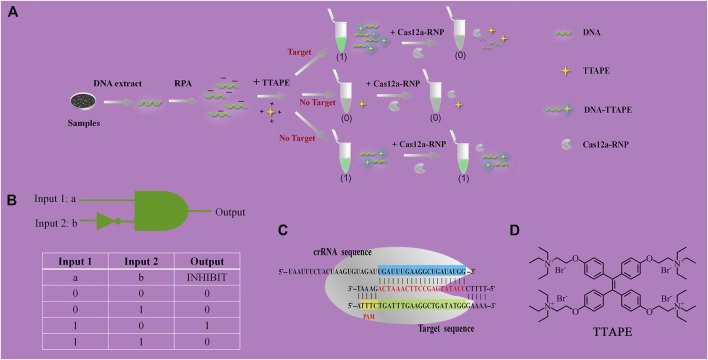
**(A)** Schematic diagram of TTAPE-CRISPR/Cas12a platform for detecting pathogenic microorganisms; **(B)** Logical gate schematic diagram and real table value; **(C)** The process of CRISPR/Cas12a to recognize specific DNA fragments of *L. pneumophila*; **(D)** Chemical structure of TTAPE.

The mechanism diagram of the detection method is shown in Figure 1A. DNA that extracted from pathogenic microorganisms is amplified by RPA. DNA with negative electricity and TTAPE with positive electricity bind non-specifically via electrostatic action, causing TTAPE to aggregate with the DNA and emit a strong fluorescent signal. However, if the sample is directly added to TTAPE after RPA, false positive results will often occur. The introduction of CRISPR/Cas12a technology into the system can eliminate false positive results and improve the reliability of the results. Upon adding Cas12a-RNP (the combination of Cas12a and crRNA), crRNA will successfully recognize the specific DNA, then Cas12a starts to randomly cut all DNA in the system, and all long-stranded DNA is cut into short-stranded DNA of 3–5 bp. As a result, aggregated TTAPE was dissociated, leading to fluorescence quenched. In contrast, if no specific DNA fragment exist, crRNA recognition fails, and the DNA chains is not cut by Cas12a. As a result, TTAPE still accumulates on DNA in large quantities, and not obvious fluorescence change can be observed. Therefore, the rapid detection of pathogenic microorganisms can be achieved visually by CRISPR/Cas12a technology combined with TTAPE.

### 2.2 Cas12a-RNP Activity Study

To explore the shear activity of Cas12a-RNP, the molecular weights of the amplified samples of three pathogenic microorganisms before and after the Cas12a-RNP treatment were tested. This process was tested by agarose gel electrophoresis. As shown in Supplementary Figure S1, before the Cas12a-RNP treatment, the amplified samples of the three pathogenic microorganisms all had larger molecular weights, with lengths of 291, 240, and 906 bp respectively. After the Cas12a-RNP treatment, a large number of DNA strands in the sample were sheared and degraded by Cas12a-RNP, and the DNA molecular weight of the amplified samples was significantly reduced. This phenomenon indicated that Cas12a-RNP could specifically recognize the three pathogenic microorganisms, and had a strong shearing activity on the DNA of the three pathogenic microorganisms.

### 2.3 Specificity Study

**FIGURE 2:**
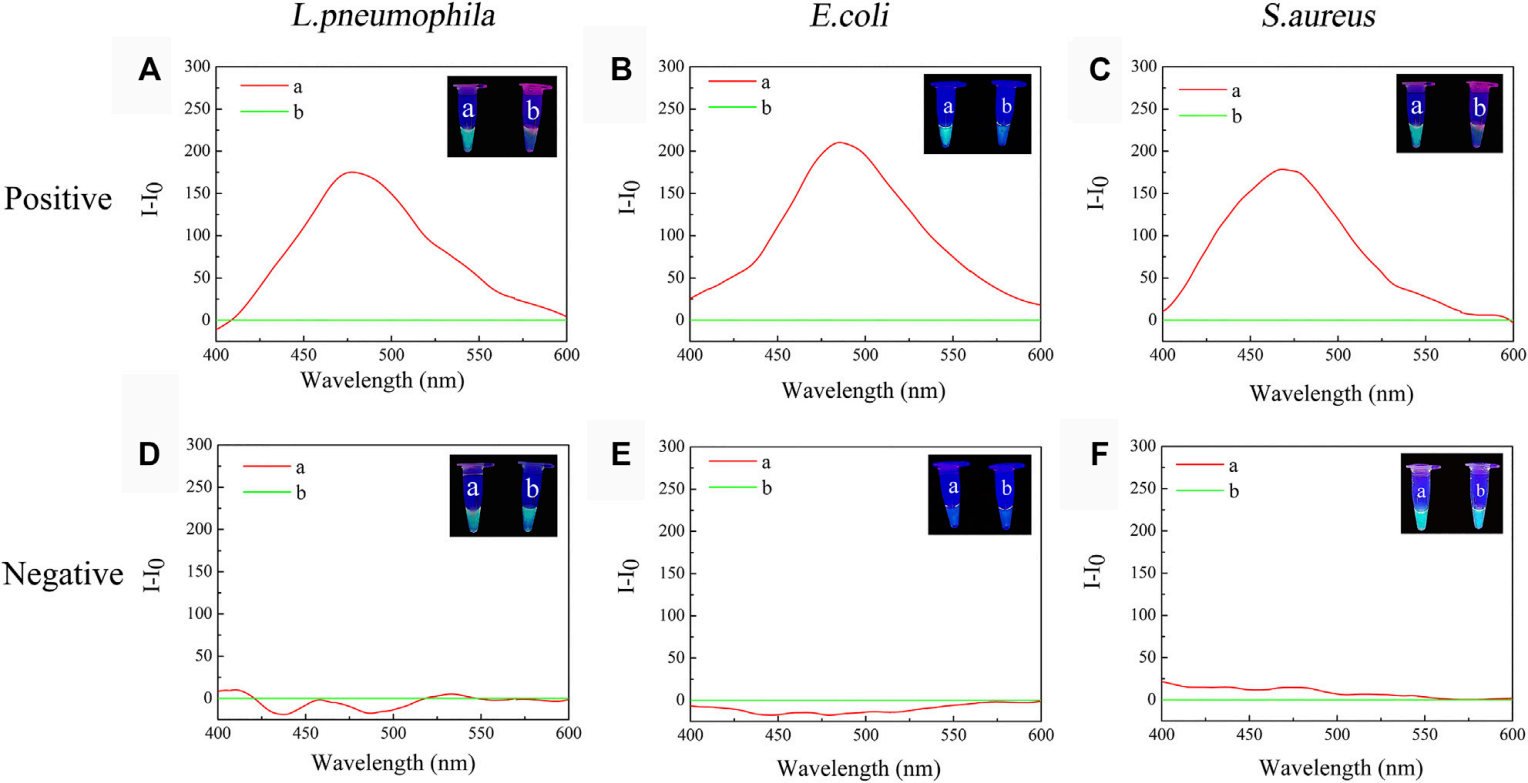
The visual discrimination results under UV irradiation (365 nm) and PL spectra (λ_ex._ = 350 nm) of **(A)** *L. pneumophila* positive sample; **(B)** *E.coli* positive sample; **(C)** *S.aureus* positive sample; **(D)** *L. pneumophila* negative sample; **(E)** *E.coli* negative sample; **(F)** *S.aureus* negative sample (a: +TTAPE, b: +TTAPE +Cas12a-RNP).

Negative samples and positive samples were treated simultaneously to determine whether the constructed TTAPE-CRISPR/Cas12a platform could achieve specific detection. In order to visualize the detection results, two identical positive samples (a, b) were prepared, the tube a was treated only by TTAPE, and the tube b was treated by TTAPE and Cas12a-RNP. After 5-min reaction, changes in fluorescence intensity in the positive sample tube was observed directly with the naked eye under ultraviolet (UV) irradiation (365 nm). However, two negative samples tube had not obvious change. As shown in Figures 2A–C represent the relative fluorescence intensity of the positive samples of *L. pneumophila, E. coli* and *S.aureus* in the presence or absence of Cas12a-RNP (the fluorescence intensity of tube b minus the fluorescence intensity of tube a). Figures 2D–E represent the relative fluorescence intensity of the negative samples of *L. pneumophila, E. coli* and *S.aureus* in the presence or absence of Cas12a-RNP. The original fluorescence intensity is shown in Supplementary Figures S2, S3, S4. The fluorescence intensity of the tube b of the positive sample of the three pathogenic microorganisms was significantly reduced compared with the tube a, while the fluorescence intensity of negative sample was not significantly change. The reason was that Cas12a-RNP identified specific DNA fragments in the positive sample and all long-stranded DNA was cut by Cas12a, leading to fluorescence quenched. The results show that the TTAPE-Cas12a detection platform can visually observe the changes in the fluorescence intensity of the samples, which can distinguish between negative and positive samples of pathogenic microorganisms.

### 2.4 Logic Gate Analysis

When tube a or tube b emits a fluorescent signal, we set it as 1, when there was no fluorescence or fluorescence was quenched, we set it as 0. Tube a was input 1, and tube b was input 2. Then, when tube a was bright and tube b was dark, the output value was represented by (1,0), which meant that the target was detected, as shown in Figures 2A–C. When tube a and tube b were both bright, the output value was represented by (1,1), which meant that no target was detected, as shown in Figures 2D,F. The reason why tubes a and b were both bright was that although there was no target, the primer dimer formed would cause TTAPE to accumulate on the DNA, causing the turn emission of TTAPE. When tube a and tube b were both dark, the output value was represented by (0,0), as shown in Figure 2E, which meant that no target was detected. Therefore, only when the logic gate signal output was (1,0), can it represent the detection of the target.

### 2.5 Method Sensitivity

**FIGURE 3:**
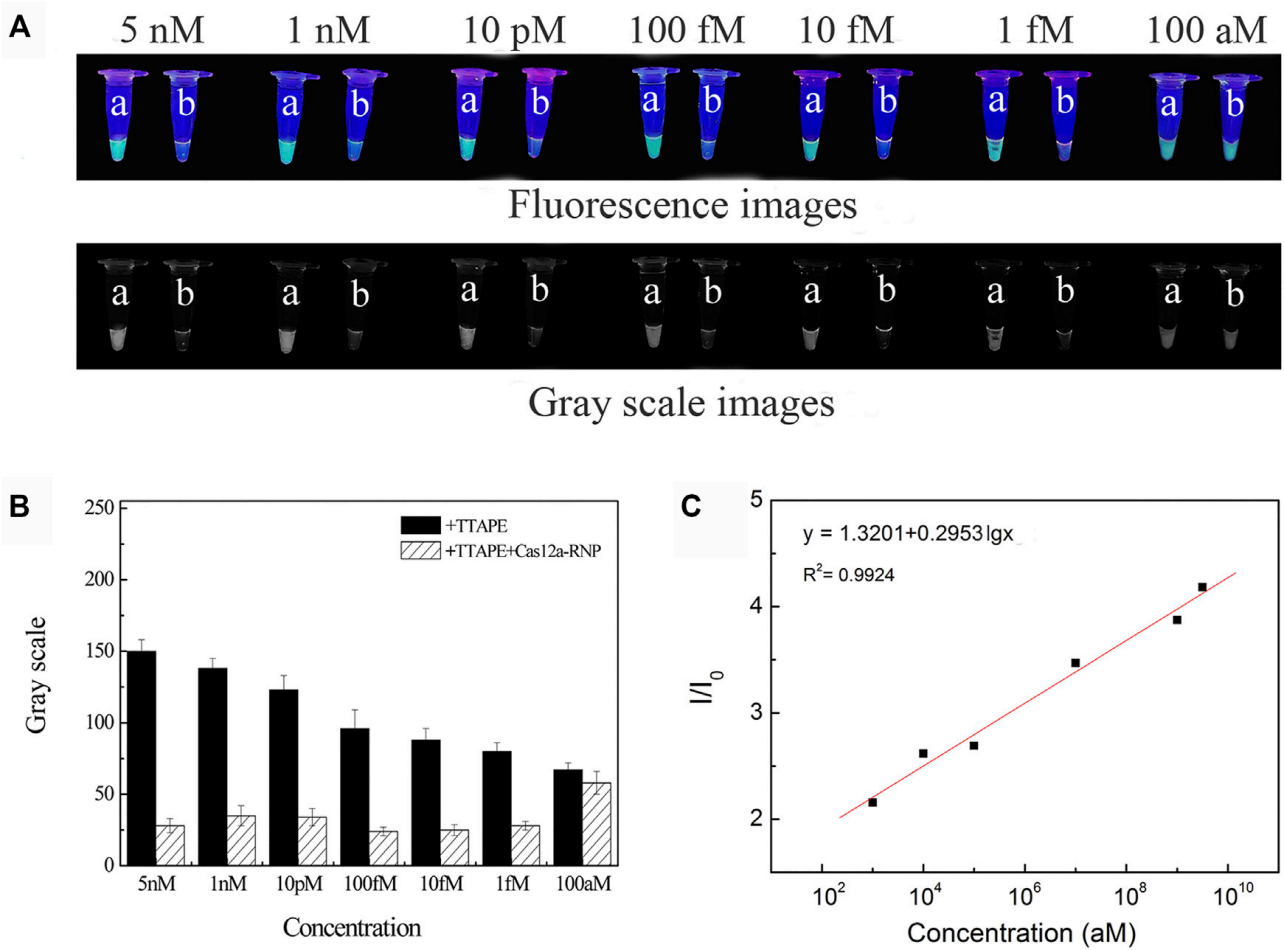
**(A)** The visual discrimination results under UV irradiation and gray scale images of *L. pneumophila* at different concentrations (a: +TTAPE, b: +TTAPE +Cas12a-RNP); **(B)** Gray value of *L. pneumophila* at different concentrations (the gray value is obtained by analyzing the gray image by image J). **(C)** Linear relationship of the fluorescence (I/I_0_) and logarithm concentration of *L. pneumophila*.

The sample of *L. pneumophila* DNA concentration of 5 nM, 1 nM, 10 pM, 100 fM, 10 fM, 1 fM, 100 aM were respectively tested (Figure 3A). Fluorescence quenching occurred in tubes b in the range of DNA concentration of 5 nM-1 fM. When the concentration is less than 1fM, it is difficult to directly observe the fluorescent quenching with the naked eye (Figure 3B). According to LOD = 3S/b, the detection limit of L. pneumophila DNA concentration is calculated to be 3 aM (Figure 3C). And as the DNA concentration decreased, the fluorescent intensity of the tube a also decreased. The concentration of the target in the system could be quantitated by the software (image J) analyzing the size of grayscale values (Figure 3B). The sensitivity of *E.coli* and *S.aureus* using the TTAPE-CRISPR/Cas12a platform (Supplementary Figures S5, S6).

### 2.6 Detection of Water Samples in the Environment

**FIGURE 4:**
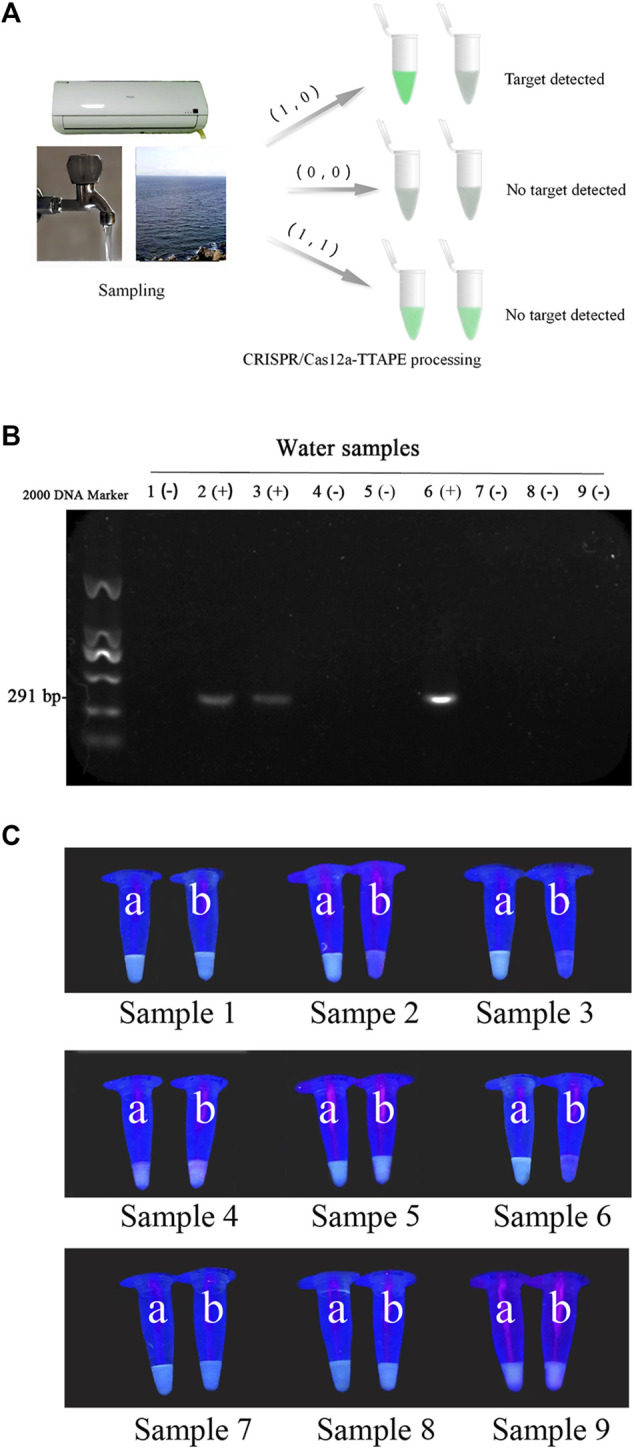
**(A)** Diagram of the TTAPE-CRISPR/Cas12a platform for detecting environmental water samples; Gel electrophoresis diagram **(B)** and the visual discrimination results under UV irradiation **(C)** of *L. pneumophila* in environmental water samples (The target DNA length is 291 bp. “+” represents samples containing *L. pneumophila*, “−” represents samples without *L. pneumophila*. 1–3: air-conditioned water samples, 4–6: lake water samples, 7–9: tap water samples; a: +TTAPE, b: +TTAPE +Cas12a-RNP).

To further verify the actual feasibility of the TTAPE-CRISPR/Cas12a detection platform, the water samples in environment were determined. A total of 3 air conditioning water samples, 3 tap water samples and 3 lake water samples were treated by the TTAPE-CRISPR/Cas12a platform, and the fluorescent change of various water samples was obtained. The gel electrophoresis was tested on 9 water samples, and the fluorescence change of various water samples was compared with the gel diagram (Figures 4B,C). The TTAPE-CRISPR/Cas12a platform was highly matched with the gel electrophoresis method. The detection results of *E.coli* and *S.aureus* water samples using the TTAPE-CRISPR/Cas12a platform and gel electrophoresis were also consistent (Supplementary Figures S7, S8). Therefore, the feasibility and universality of the TTAPE-CRISPR/Cas12a platform are further verified.

## 3 Materials and Methods

### 3.1 Materials

*L. pneumophila* (ATCC 33152), *E.coli* (ATCC 25922), *S.aureus* (CMCC 26003) were from purchased Shanghai Bioresource Collection Center. BCYE solid Culture, GVPC liquid Culture, LB Culture were purchased from HuanKai Biology Co., Ltd. (Guangdong, China). Bacterial Genomic DNA Extraction Kit were purchased from Tiangen Biochemical Technology Co., Ltd. (Beijing, China). TwistAmp^®^ Basic RPA kit were purchased from TwistDx Inc. (United Kingdom). RPA primers were purchased from the Shanghai Sangon Biological Science & Technology Company (Shanghai, China). Agarose, SYBR Green nucleic acid dye, DNA Marker were purchased from ThermoFisher Scientifific. CRISPR-Cas12a protein, crRNA were from Guangzhou Bio-Lieesci Co., Ltd. Air-conditioned water, lake water, tap water were from Dongguan University of Technology (Dongguan, China).

### 3.2 Bacterial Culture

The standard strain of *L. pneumophila* (ATCC 33152), *E.coli* (ATCC 25922) and *S.aureus (*CMCC 26003) was purchased from the Shanghai Collection Center. After *L. pneumophila* strain was inoculated into the BCYE solid culture, culturing in a CO_2_ incubator at 37°C for 3–5 days, a single colony was picked out into GVPC liquid culture, cultured in a thermostatic shaker at 37°C for 3–5 days. The *E. coli* and *S.aureus* strain were inoculated into the LB solid culture, it was cultured overnight in thermostatic incubator at 37°C, a single colony was picked out and placed in the LB liquid culture, and cultured overnight in a thermostatic shaker at 37°C. Obtained purified positive samples of three pathogenic microorganisms. The growth and concentration of the bacteria was measured by an ultraviolet spectrophotometer to measure OD600.

### 3.3 Design and Synthesis of RPA Primer Pair and crRNA

Designed the RPA amplification primer pair of three pathogenic microorganism on the NCBI database and Primer 5 software. The primer pair was synthesized by Sangon Biotech. The crRNA sequence was designed and synthesized by Guangzhou Bio-Lieesci Co., Led. The RPA primer pairs and crRNA sequence of pathogenic microorganism were shown in Supplementary Table S2.

### 3.4 DNA Extraction and Amplification

ddH_2_O was used as a negative sample, and pathogenic microorganism was used as a positive sample. Extract the genomic DNA of ddH_2_O and pathogenic microorganism by bacterial genomic DNA extraction kit. The OD 260/OD 280 of the DNA extract was between 1.6 and 1.8. The extract was stored in a refrigerator at −20°C. The DNA fragment of pathogenic microorganism was amplificated by wistAmp^®^ Basic RPA kit at 39°C for 15 min. The entire system was 50 μl, which made the DNA fragments enriched in a large amount. RPA amplification system was shown in Supplementary Table S3.

### 3.5 Cas12a-RNP Activity Study

Two RPA products of pathogenic microorganism were put into two tubes (a, b)*.* Added ddH_2_O to tube a, 100 nM Cas12a, 120 nM crRNA and 1*buffe to tube b. After reacting at 37°C for about 5 min, both were subjected to gel electrophoresis by an electrophoresis apparatus.

### 3.6 TTAPE Optimization

The fluorescence intensity changes in the absence of TTAPE and different concentrations of TTAPE were tested, and the detection concentration of TTAPE was optimized. Using *L. pneumophila* as an optimized sample. First, in the absence of TTAPE, there was no appearance or change of fluorescence in the system (Supplementary Figure S9A). When the concentration of TTAPE was low, it was difficult to see the fluorescence of the positive sample itself under low concentration of DNA (Supplementary Figure S9B). When the concentration of TTAPE was high, due to the aggregation of TTAPE itself, it was difficult to see whether the fluorescence is quenched at low concentrations of DNA (Supplementary Figure S9C). The quenching phenomenon of TTAPE could be observed obviously between 40 and 80 μM (Supplementary Figure S9D). When the concentration of TTAPE was 50 μM, the lowest detection limit could be reached. Decreased or increased the concentration of TTAPE would reduce the sensitivity of detection. The optimized system of TTAPE at different concentrations is shown in Supplementary Table S1.

### 3.7 Specificity Study

Two RPA products of positive samples were put into two tubes (a, b)*.* 50 μM TTAPE was added to tube a, 100 nM Cas12a, 120 nM crRNA and 1*buffer was added to tube b after added 50 μM TTAPE (Supplementary Table S4). Negative sample was treated in the same method as positive samples. After 5 min of the reaction, the changes in the fluorescent intensity in the four sample tubes were directly observed with the naked eye under UV irradiation. And their fluorescence intensity was measured using a fluorescent spectrophotometric.

### 3.8 Sensitivity Study

Pathogenic microorganism samples with DNA concentrations of 5 nM, 1 nM, 10 pM, 100 fM, 10 fM, 1 fM, and 100 aM were prepared. After RPA amplification, the RPA products of different DNA concentrations are processed according to the above method (3.7), and then observed their fluorescence intensity under UV irradiation.

### 3.9 Water Sample Detection

3 air-conditioned water samples, 3 lake water samples and 3 tap water samples of 500 ml each were prepared. DNA extraction of microorganisms in the water obtains the water samples to be detected. After RPA amplification, the amplification production of water samples were treated according to the above method (3.7), the fluorescent maps of various water samples were obtained. The gel electrophoresis detection of the water samples was conducted, and the fluorescence was compared to gel electrophoresis.

## 4 Conclusion

In conclusion, the TTAPE-CRISPR/Cas12a Logic Gate detection platform was developed based on the aggregation-induced emission material TTAPE and the random shearing feature of the CRISPR/Cas12a. This platform can detect pathogenic microorganism in the environment, Including *L. pneumophila, E. coli* and *S.aureus* specifically, quickly and visually, and the detection limit of pathogenic microorganism can reach 3 aM. The TTAPE-CRISPR/Cas12a platform has the advantages of speedy detection, high specificity, simple operation, and only one simple kit. It is expected to be applied to the actual on-site detection of environmental water samples. In the outbreak of disease caused by pathogenic microorganisms, this method can be used to rapidly detect the source of infection to reduce the threat of pathogenic microorganisms to human life and health.

## Data Availability

The original contributions presented in the study are included in the article/Supplementary Material, further inquiries can be directed to the corresponding authors.
