# Supplementary figures and images for: CRISPR/Cas12a-Assisted Visual Logic-Gate Detection of Pathogenic Microorganisms Based on Water-Soluble DNA-Binding AIEgens
Source: Front Chem. 2022 Jan 14;9:801972. doi: 10.3389/fchem.2021.801972 (PMC8795674; doi:10.3389/fchem.2021.801972)

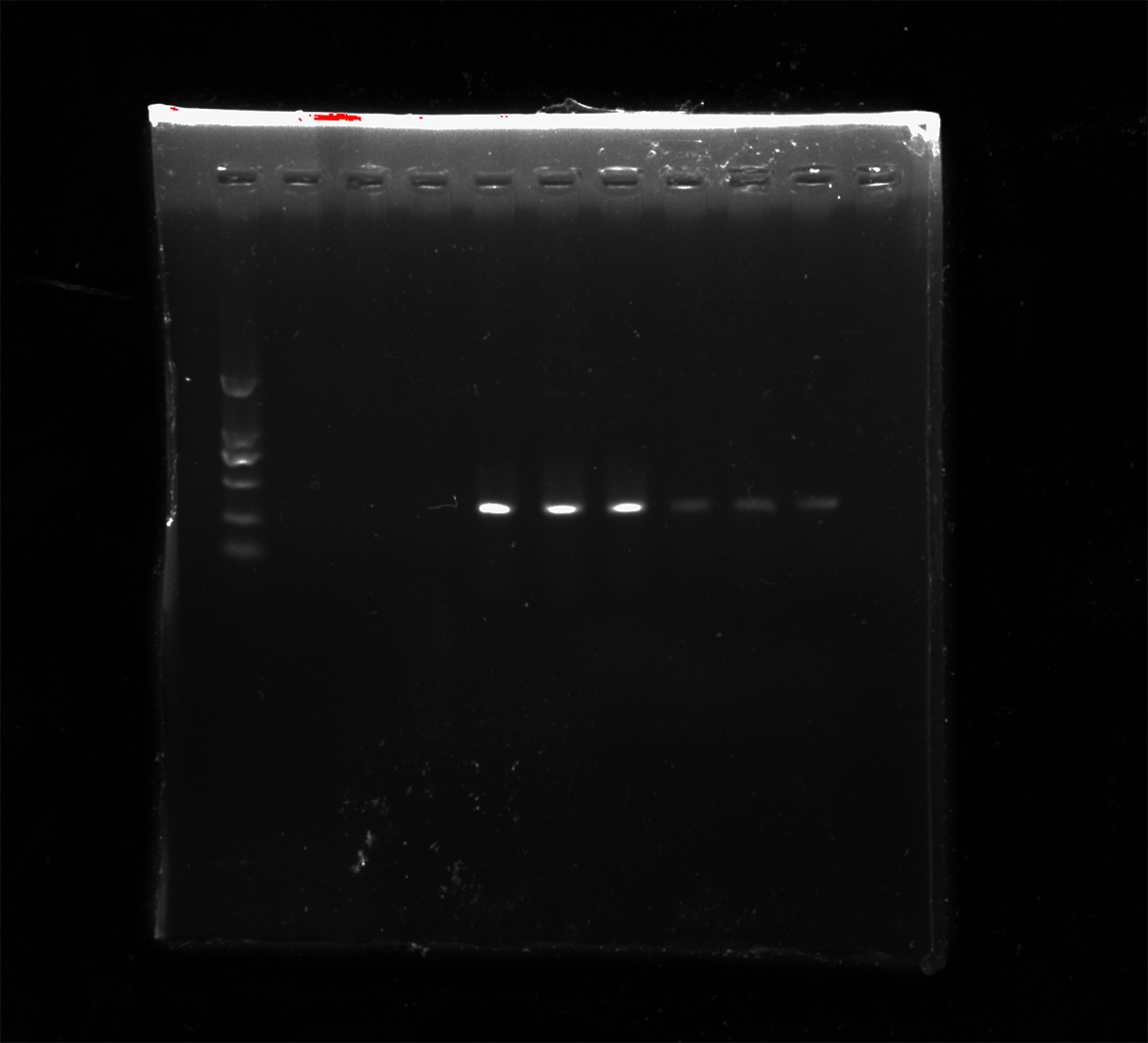

Supplement: Supplementary file 1 [file DataSheet1.ZIP › Original date/Original gels/Fig S1-A.tif]

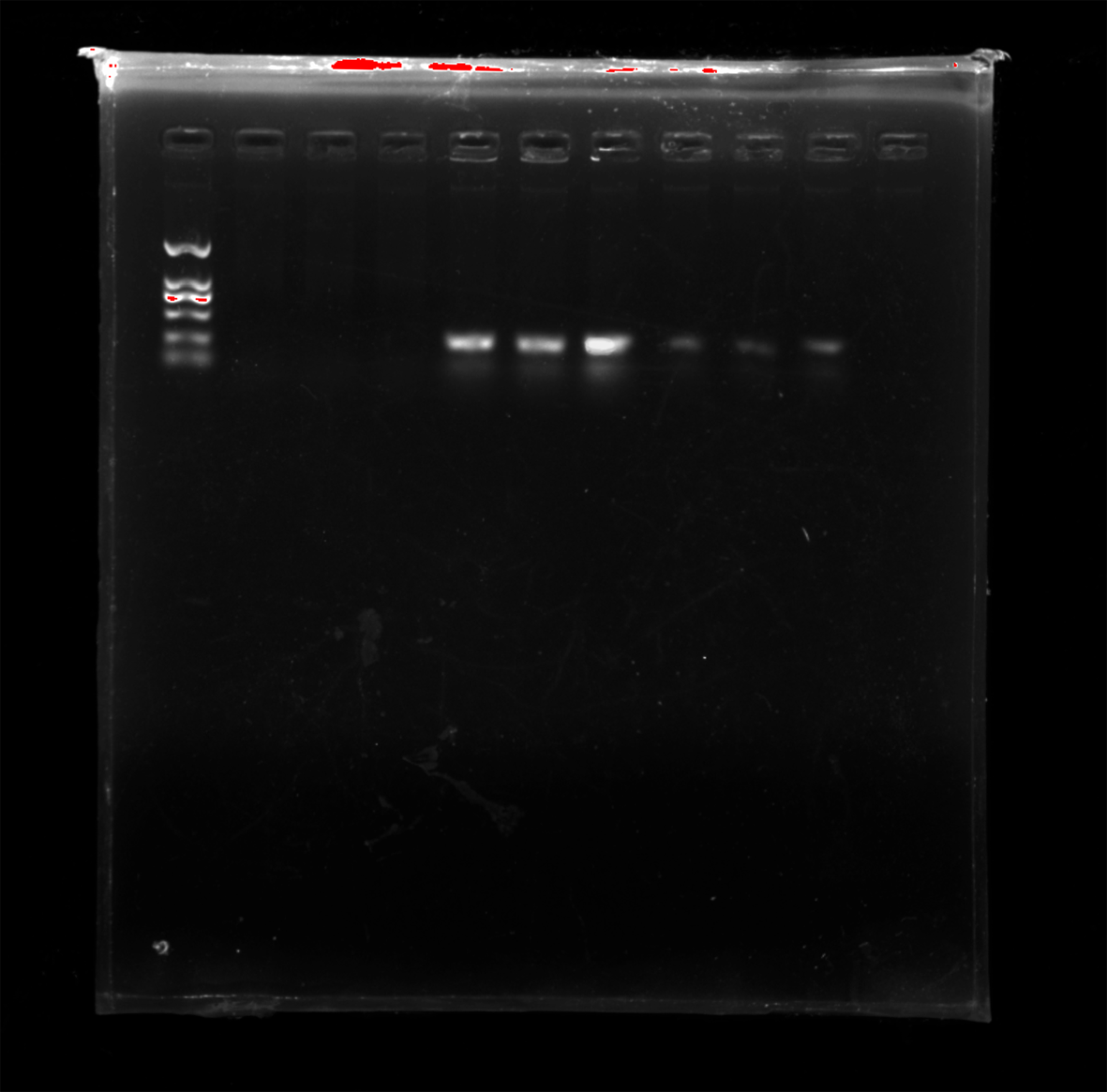

Supplement: Supplementary file 1 [file DataSheet1.ZIP › Original date/Original gels/Fig S1-B.tif]
