# Supplementary material for: CRISPR/Cas12a-Assisted Visual Logic-Gate Detection of Pathogenic Microorganisms Based on Water-Soluble DNA-Binding AIEgens
Source: Front Chem. 2022 Jan 14;9:801972. doi: 10.3389/fchem.2021.801972 (PMC8795674; doi:10.3389/fchem.2021.801972)
Supplement: Supplementary file 2 [file DataSheet2.docx]

**Supporting Information**

**CONTENT**

Figure S1. ......................................................................................................................1

Figure S2. ......................................................................................................................2

Figure S3. ......................................................................................................................3

Figure S4. ......................................................................................................................4

Figure S5. ......................................................................................................................5

Figure S6. ......................................................................................................................6

Figure S7. ......................................................................................................................7

Figure S8. ......................................................................................................................8

Figure S9. ......................................................................................................................9

Table S1. ......................................................................................................................10

Table S2. ......................................................................................................................11

Table S3. ......................................................................................................................11

Table S3. ......................................................................................................................12


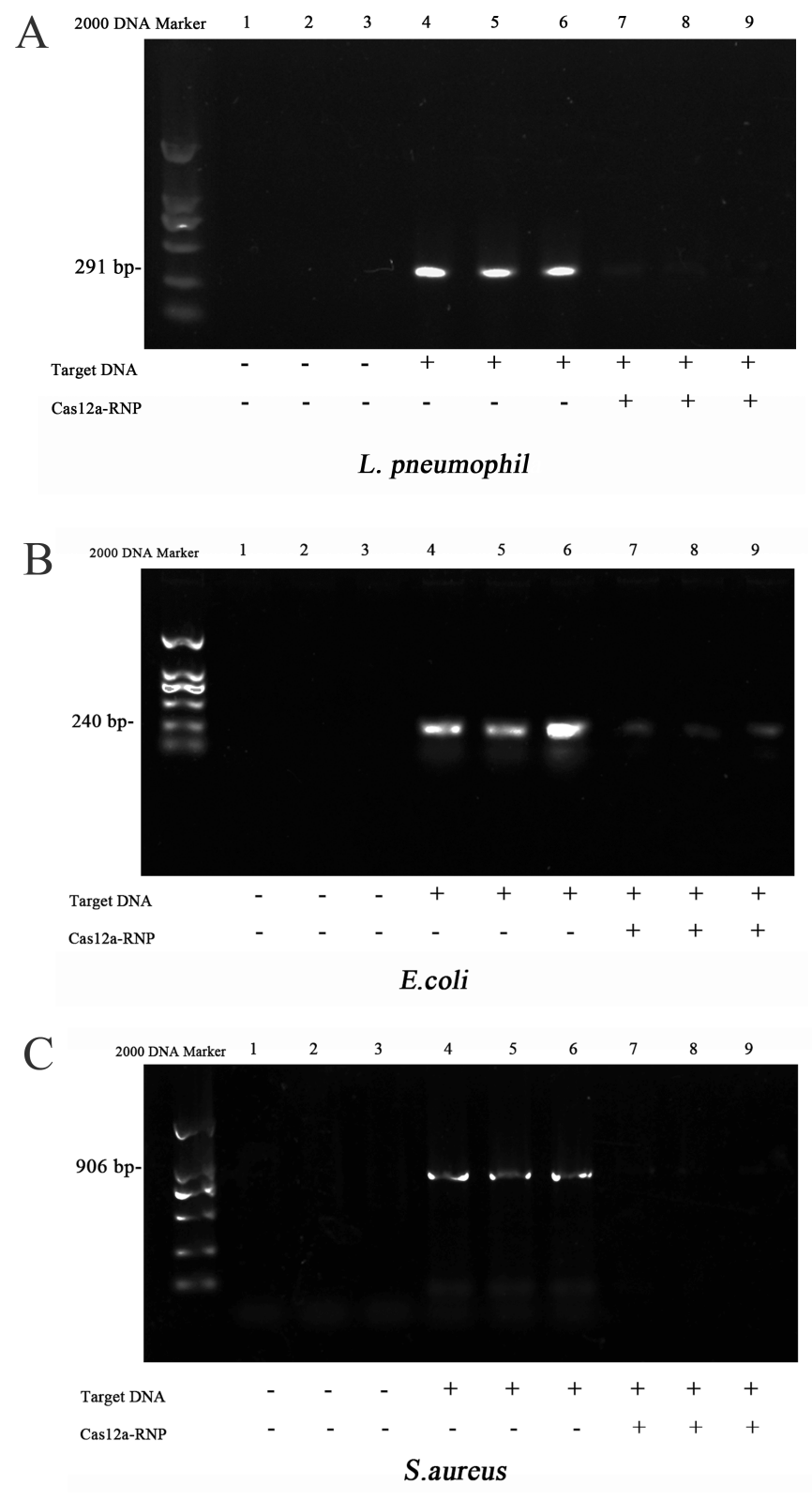


Figure S1. Cas12a-RNP activity detection of *L. pneumophila* (A), *E.coli* (B) and *S.aureus* (C). (Cas12a-RNP reaction for 5 min. “+” represents samples containing Target DNA/Cas12a-RNP, “-” represents samples without Target DNA/Cas12a-RNP. After 5 minutes of Cas12a-RNP reaction, the samples were subjected to agar gel electrophoresis.)


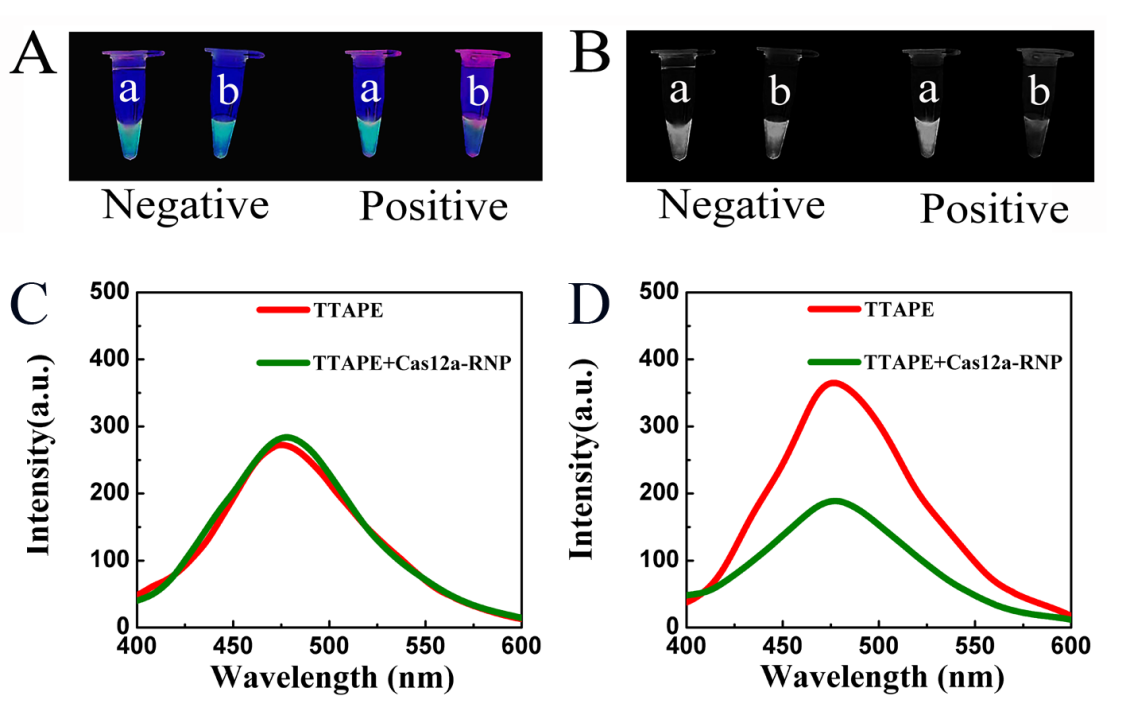


Figure S2. Fluorescence images (A) and gray scale images (B) of negative sample and positive sample of *L. pneumophila* (a: +TTAPE, b: +TTAPE +Cas12a-RNP); Fluorescence change spectrums of negative samples (C) and positive samples (D) of *L. pneumophila*.


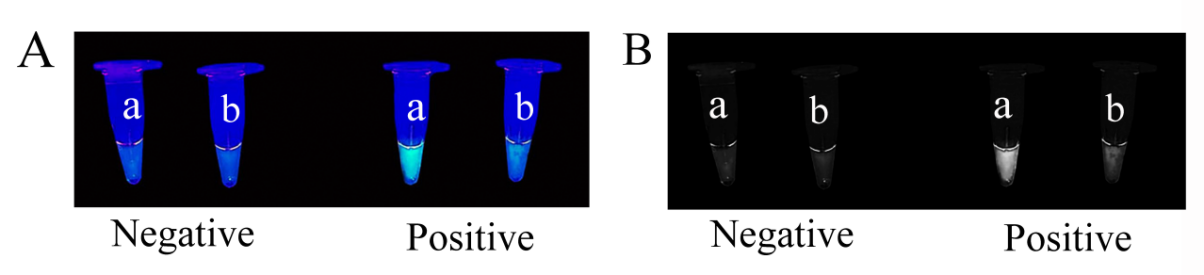


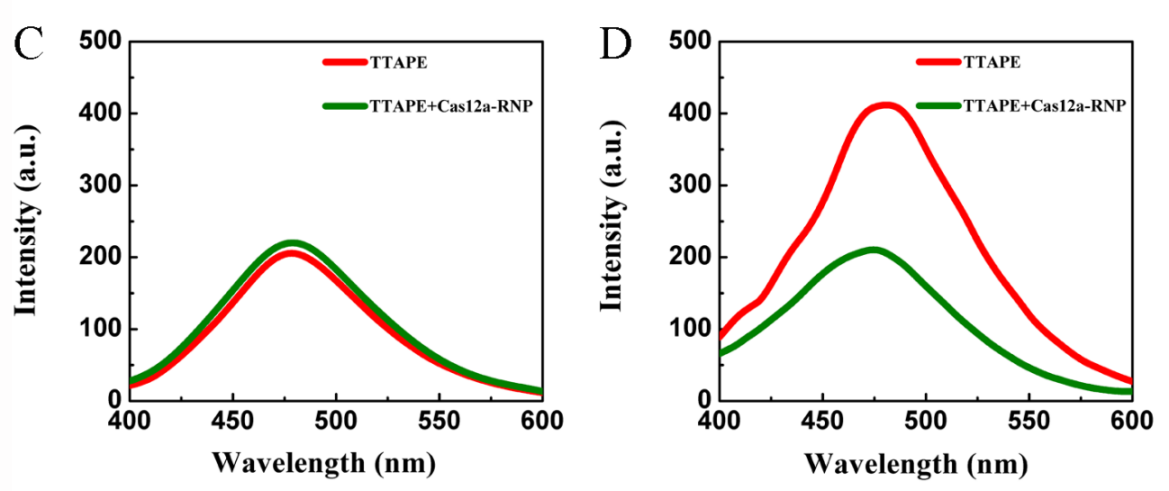


Figure S3. Fluorescence images (A) and gray scale images (B) of negative sample and positive sample of *E.coli* (a: +TTAPE, b: +TTAPE +Cas12a-RNP); Fluorescence change spectrums of negative samples (C) and positive samples (D) of *E.coli*.


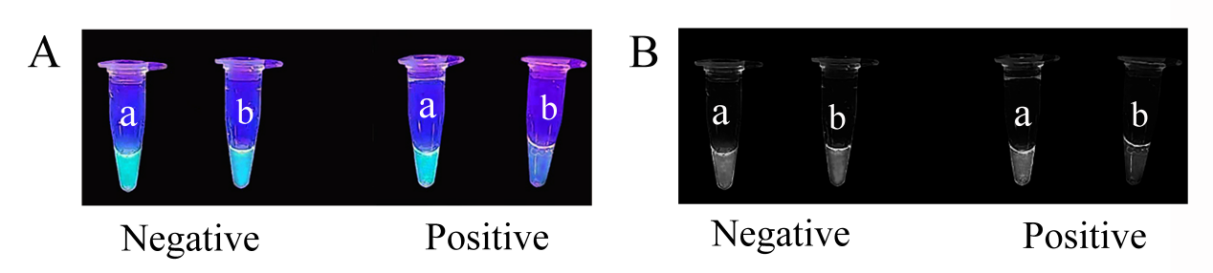


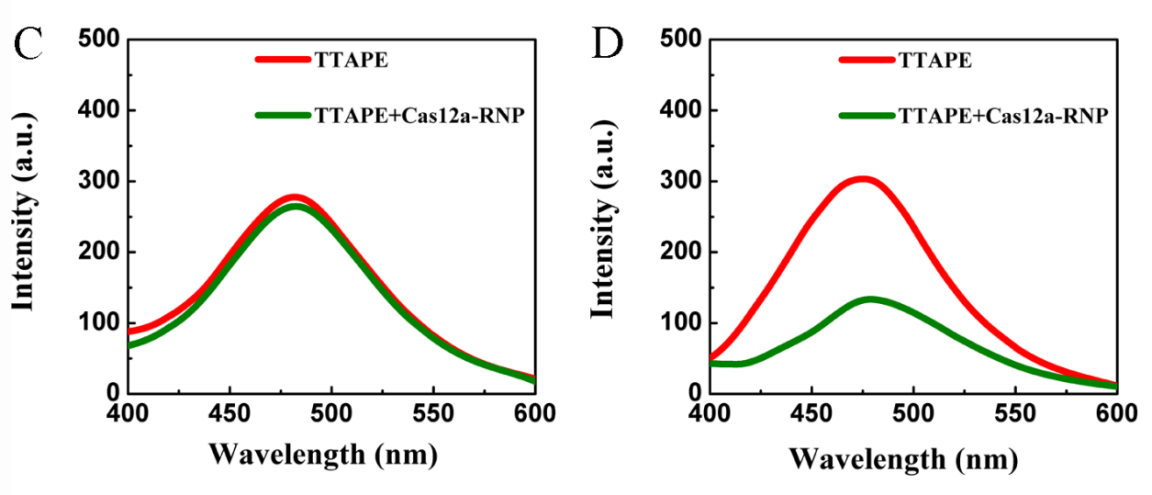


Figure S4. Fluorescence images (A) and gray scale images (B) of negative sample and positive sample of *S.aureus* (a: +TTAPE, b: +TTAPE +Cas12a-RNP); Fluorescence change spectrums of negative samples (C) and positive samples (D) of *S.aureus.*


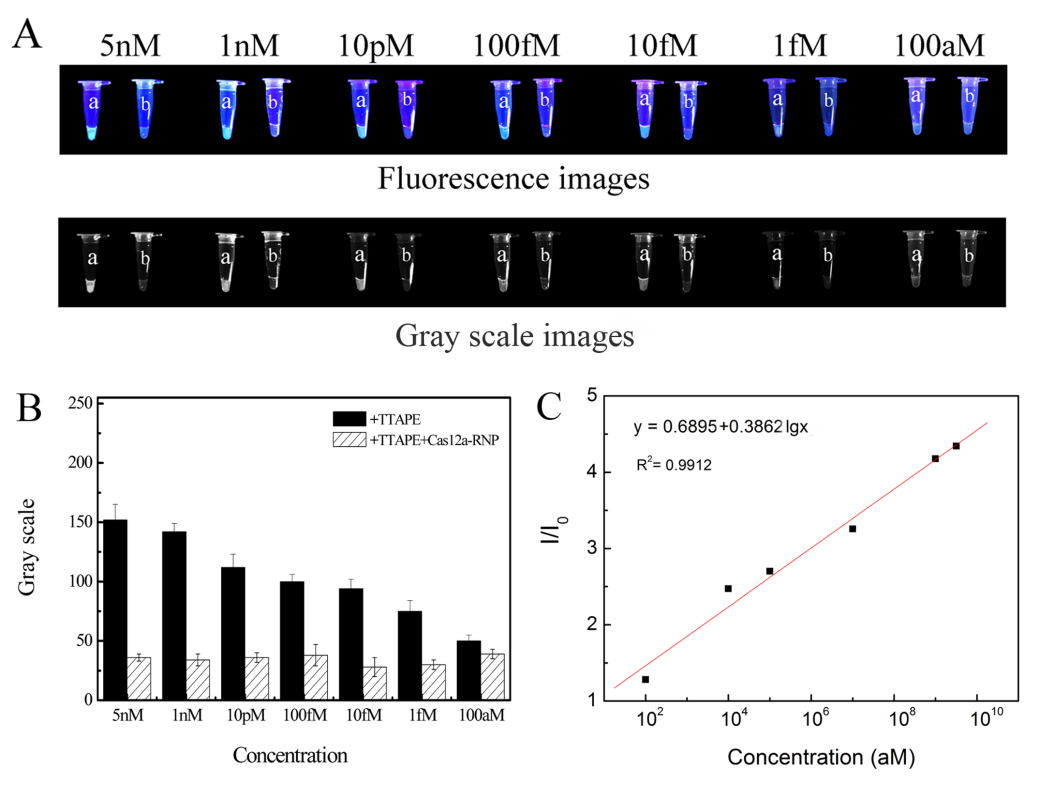


Figure S5. (A) Fluorescence images and gray scale images of *E.coli* at different concentrations (a: +TTAPE, b: +TTAPE +Cas12a-RNP); (B) Gray value of *E.coli* at different concentrations. (C) Linear relationship of the fluorescence (I/ I_0_) and logarithm concentration of *E.coli*.


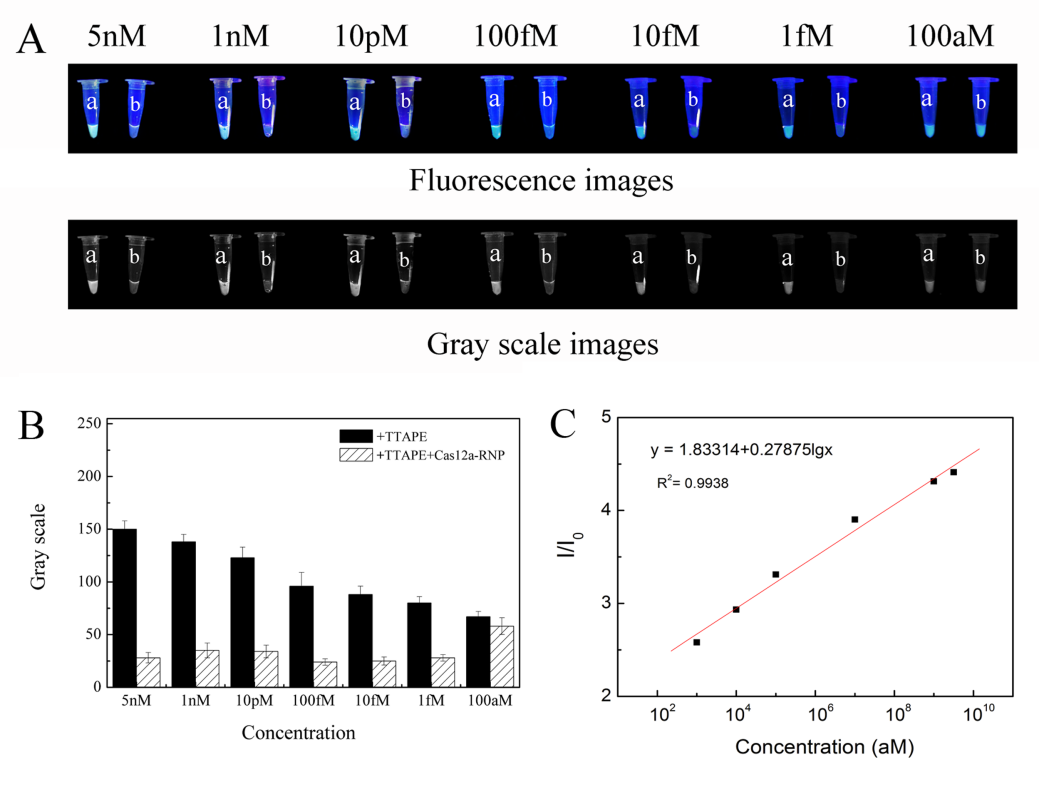


Figure S6. (A) Fluorescence images and gray scale images of *S.aureus* at different concentrations (a: +TTAPE, b: +TTAPE +Cas12a-RNP); (B) Gray value of *S. aureus* at different concentrations. (C) Linear relationship of the fluorescence (I/ I_0_) and logarithm concentration of *S.aureus*.


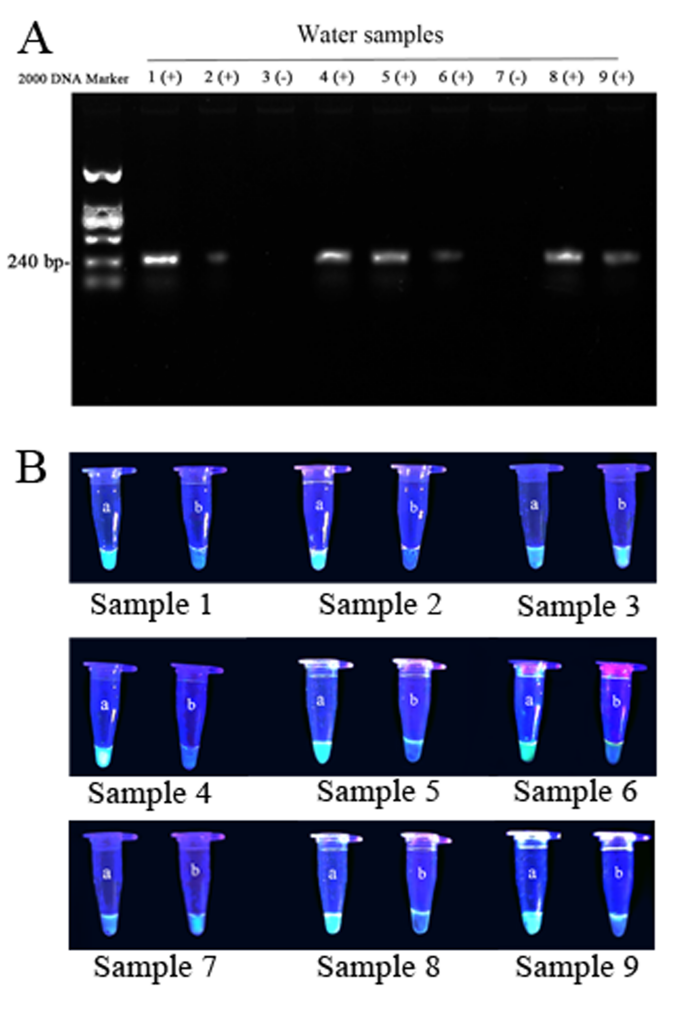


Figure S7. Gel electrophoresis diagram (A) and Fluorescence images (B) of *E.coli* in environmental water samples (“+” represents samples containing *E.coli*, “-” represents samples without *E.coli*. 1-3: air-conditioned water samples, 4-6: lake water samples, 7-9: tap water samples; a: +TTAPE, b: +TTAPE +Cas12a-RNP)


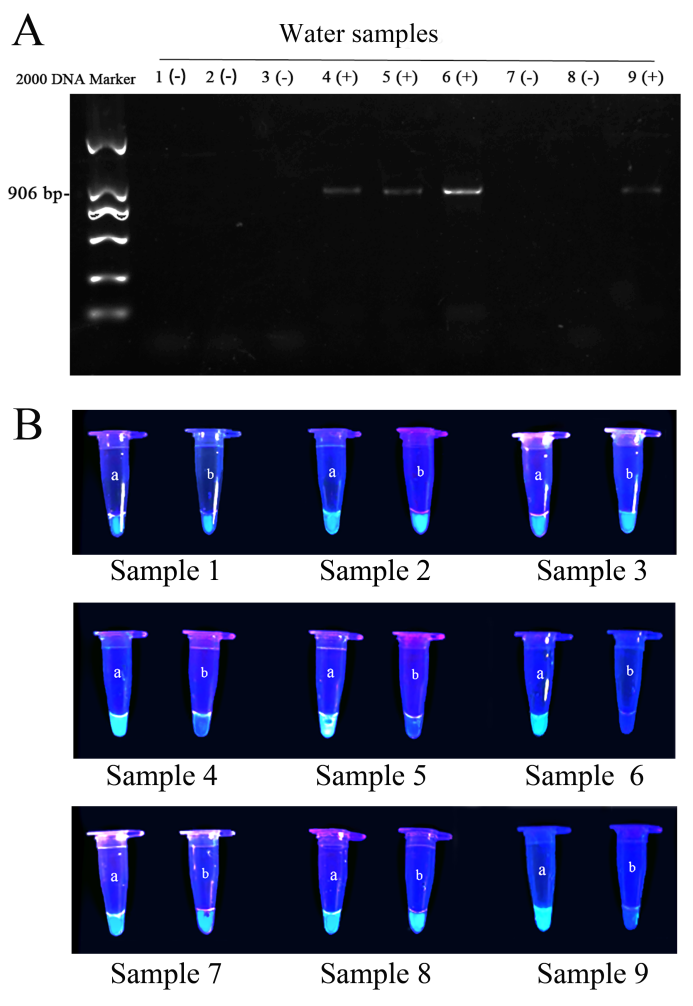


Figure S8. Gel electrophoresis diagram (A) and Fluorescence images (B) of *S.aureus* in environmental water samples (“+” represents samples containing *S.aureus*, “-” represents samples without *S.aureus*.1-3: air-conditioned water samples, 4-6: lake water samples, 7-9: tap water samples; a: +TTAPE, b: +TTAPE +Cas12a-RNP)


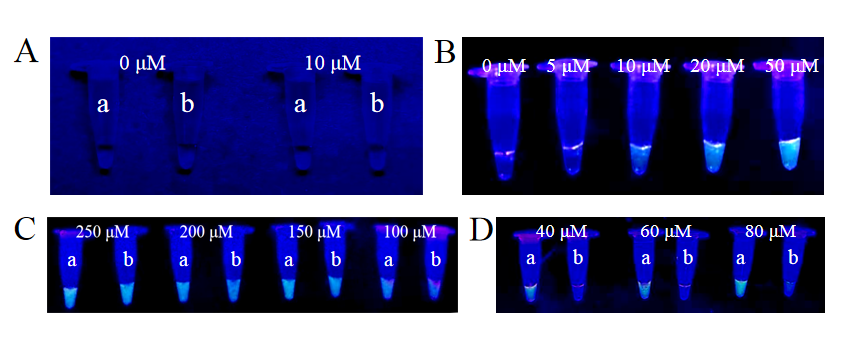


Figure S9. (A) The fluorescence changes of the *L. pneumophila* samples in the absence of TTAPE; (B) Fluorescence of *L. pneumophila* samples under low-concentration of TTAPE; (C) Fluorescence of *L. pneumophila* samples under high-concentration of TTAPE; (D) Fluorescence of *L. pneumophila* samples when the TTAPE concentration was 40 μM, 60 μM, and 80 μM.

Table S1. Different concentrations of TTAPE optimized system

|  | **ddH_2_O** | **Template（1 pM）** | **TTAPE (5 μL)** | **Cas12a (1 μM)** | **1*buffer** | **crRNA (1.2 μM)** |
| --- | --- | --- | --- | --- | --- | --- |
| 1 | 17.5 μL | 2.5 μL | 0 μM | 0 μL | 0 μL | 0 μL |
| 2 | 10 μL | 2.5 μL | 0 μM | 2.5 μL | 2.5 μL | 2.5 μL |
| 3 | 17.5 μL | 2.5 μL | 5 μM | 0 μL | 0 μL | 0 μL |
| 4 | 17.5 μL | 2.5 μL | 10 μM | 0 μL | 0 μL | 0 μL |
| 5 | 10 μL | 2.5 μL | 10 μM | 2.5 μL | 2.5 μL | 2.5 μL |
| 6 | 17.5 μL | 2.5 μL | 20 μM | 0 μL | 0 μL | 0 μL |
| 7 | 17.5 μL | 2.5 μL | 50 μM | 0 μL | 0 μL | 0 μL |
| 8 | 17.5 μL | 2.5 μL | 250 μM | 0 μL | 0 μL | 0 μL |
| 9 | 10 μL | 2.5 μL | 250 μM | 2.5 μL | 2.5 μL | 2.5 μL |
| 10 | 17.5 μL | 2.5 μL | 200 μM | 0 μL | 0 μL | 0 μL |
| 11 | 10 μL | 2.5 μL | 200 μM | 2.5 μL | 2.5 μL | 2.5 μL |
| 12 | 17.5 μL | 2.5 μL | 150 μM | 0 μL | 0 μL | 0 μL |
| 13 | 10 μL | 2.5 μL | 150 μM | 2.5 μL | 2.5 μL | 2.5 μL |
| 14 | 17.5 μL | 2.5 μL | 100 μM | 0 μL | 0 μL | 0 μL |
| 15 | 10 μL | 2.5 μL | 100 μM | 2.5 μL | 2.5 μL | 2.5 μL |
| 16 | 17.5 μL | 2.5 μL | 40 μM | 0 μL | 0 μL | 0 μL |
| 17 | 10 μL | 2.5 μL | 40 μM | 2.5 μL | 2.5 μL | 2.5 μL |
| 18 | 17.5 μL | 2.5 μL | 60 μM | 0 μL | 0 μL | 0 μL |
| 19 | 10 μL | 2.5 μL | 60 μM | 2.5 μL | 2.5 μL | 2.5 μL |
| 20 | 17.5 μL | 2.5 μL | 80 μM | 0 μL | 0 μL | 0 μL |
| 21 | 10 μL | 2.5 μL | 80 μM | 2.5 μL | 2.5 μL | 2.5 μL |

Table S2. RPA amplification primers and crRNA sequence

| **Name** | | **Sequence (5’-3’)** |
| --- | --- | --- |
| *L.pneumophila* | Primer-F | ATTTGCGGCACAGTAACATGGAATAGTATTTC |
|  | Primer-R | TTGTTGATACTCAGTGCAGACCTGACCAGATG |
|  | crRNA | UAAUUUCUACUAAGUGUAGAUUGAUUUGAAGGCUGAUAUGG |
| *E. coli* | Primer-F | CGAACGGTAACAGGAACGAGC |
|  | Primer-R | CCTCTCAGACCAGCTAGGGA |
|  | crRNA | UAAUUUCUACUAAGUGUAGAUGCGUGGACUACCAGGGUAUC |
| *S. aureus* | Primer-F | CGGATCGTAAAACTCTGTTATTAGGGAAG |
|  | Primer-R | GAAGGCTCTATCTCTAGAGTTGTCAAAG |
|  | crRNA | UAAUUUCUACUAAGUGUAGAUCAAUGACCCUCCACGGUUGA |

Table S3. RPA amplification system

| **Composition** | **Volume（50 μL）** |
| --- | --- |
| Primer A (10 μM) | 2.4 μL |
| Primer B (10 μM) | 2.4 μL |
| Primer Free Rehydration buffer | 29.5 μL |
| **Template** and ddH_2_O to | 13.2 μL |
| MgOAc (280 mM) | 2.5 μL |

Table S4. TTAPE-CRISPR/Cas12a Platform detection system

| **Composition** | | **Volume（50 μL）** |
| --- | --- | --- |
| Tube a | TTAPE (200-400 μM) | 10 μL |
|  | Cas12a (1 μM) | 5 μL |
|  | cr RNA (1.2 μM) | 5 μL |
|  | 1*buffer | 5 μL |
|  | Target DNA amplification product or ddH_2_O | 10 μL |
|  | ddH_2_O | 15 μL |
| Tube b | TTAPE (200-400 μM) | 10 μL |
|  | Target DNA amplification product or ddH_2_O | 10 μL |
|  | ddH_2_O | 30 μL |
